# A community call to action: mitigating COVID pandemic’s impact on mental health

**DOI:** 10.2217/fvl-2021-0169

**Published:** 2022-03-17

**Authors:** Wahid Ullah, Muhammad Ilyas, Mukhtar Alam, Jong Bhak, Peter J Tonellato

**Affiliations:** ^1^Centre for Omic Sciences, Islamia College University Peshawar, 25120, Pakistan; ^2^Department of Bioengineering, University of Engineering and Applied Sciences, Swat, 19060, Pakistan; ^3^Dean Faculty of Sciences, University of Swabi-23430, Pakistan; ^4^School of Life Sciences, Ulsan National Institute of Science & Technology (UNIST), Ulsan, 44919, South Korea; ^5^School of Medicine, University of Missouri, Columbia, MO 65212, USA

**Keywords:** COVID-19, developing countries, global, healthcare, mental health, social network, stress, suicides, vaccines

After originating in Wuhan, China, the secondary epicenters of the COVID-19 pandemic spread rapidly to other parts of China and around the world. The number of those infected by SARS-CoV-2 will surpass 250 million and will likely cause more than 6 million fatalities [1]. In the absence of blanket vaccination or reliable treatment regime, large-scale mitigation steps (including social distancing) and some level of mandated lockdown, impose enormous economic costs on individuals, companies, cities, regions and nation states. All countries will experience, and eventually overcome the negative impact of COVID-19, however, countries already poor in economic development and having even poorer scientific and public health infrastructure, are likely to face harsher direct impact. The vulnerability of such countries will be exacerbated by lack of mobilization of resources sufficient for a robust response, resulting in a prolonged period of adverse consequences. Lack of prompt, robust and adequate response to overcome the pandemic quickly will entail long periods of isolation, loss of normal human interaction and usual socialization necessary for general mental wellness. We hereby, issue a community call to action to marshal existing culturally important neighborhood, local and regional social networks to promote measures to intervene in favor of those who are most vulnerable to mental distress.

Developing countries with less than adequate economic and physical infrastructure and meager resources are unable to provide, deliver and manage high quality health services needed to quickly respond to epidemics. In addition, weak medical facilities, non availability of specialized medical personnel and strong research infrastructure reduce the options to rapidly address unique COVID-19 etiology and transmission. Precision targeting of individuals via genetic biomarkers effective in treatment prognosis; rapid, high-throughput on-site SARS-CoV-2 and antibody testing; and sophisticated means of case tracking required for rapid and targeted mitigation response to virus breakouts are highly technology dependent. For example, Pakistan’s response to the pandemic includes very high levels of social distancing compliance, excellent case reporting and a large network of community social networks that help identify individuals with COVID-19 symptoms. However, Pakistan, like many other countries, does not have the extensive and expensive network of isolation, testing and ICU biotechnology required to rapidly and fully respond to a country-wide epidemic [2].

Societies face a plethora of complications accompanying the spread of SARS-CoV-2 combined with relatively slower and limited technological response. Furthermore, the impact of lockdowns and social restrictions required to mitigate the epidemic’s spread has not only extended individuals’ isolation time but has also resulted in extended risks to mental health and vulnerability to anxiety. Well-resourced localities are rapidly responding to these risks as demonstrated by Brigham and Women’s Hospital’s (a Harvard Medical School affiliate in MA, USA) robust response to mental health risk through their website and newsletter [3]. Emerging economies already have poor containment efforts and healthcare facilities. Behavioral health systems in such countries are not fully prepared for the secondary or tertiary waves of anxiety-induced adverse reactions to COVID-19.

Here, we share our concerns with regards to the effects of the SARS-CoV-2 pandemic and the subsequent severe negative impact on the mental health of susceptible individuals both with and without the virus or the disease. Those susceptible to the most severe mental stress include asymptomatic and symptomatic SARS-CoV-2-positive individuals and their families, health workers, essential services providers and those whose living conditions put them at high risk of contracting the virus (e.g., prisoners, high density urban populations and similar communities). Such individuals or groups are all exposed to the atmosphere of fear and uncertainty prevailing in the society compounded by what appears in the social media. Evidence of postpandemic complications in anticipation of secondary/tertiary waves are already emerging, sometimes in extreme forms of suicides that may be directly related to the COVID-19 pandemic stress. A number of COVID-19 suicide cases have been reported which are turning the media’s attention. About 70 COVID-19 suicide cases have happened due to fear of COVID-19 infection, financial crisis, loneliness, work-related stress, lockdowns etc. [4]. Similarly, the rate of suicide mortality is supposed to be increased in Bangladesh [5]. Despite the lack of comprehensively collected and managed data, recurring reports of tragedies caused by post-SARS-CoV-2-positive tests have been reported from around the world [6]. Responding to the emerging situation the People’s Republic of China recently initiated an emergency program to provide targeted psychological care to COVID-19 patients, healthcare workers, SARS-CoV-2-infected individuals, people in isolation or quarantine and the families of affected people [7].

Tested and tried conventional measures practiced to mitigate stress-related anxiety and extreme adverse responses such as suicide, have been proposed as possibly the best measures to mitigate COVID-19 pandemic-related stress [2]. Social networking is critical for a sense of well-being, though the primary COVID-19 pandemic mitigation strategy of social distancing is contrary to this important human requirement. Social networking can be maintained to a certain degree through technological means such as Facebook, Zoom and WhatsApp applications. There is a tendency in electronic and social media to over-emphasize extreme negative predictions of the pandemic. Rational and responsible steps that avoid unnecessary pessimism both during and after the pandemic will reduce the likelihood of a COVID-19-related surge of mental health problems. For example, in Pakistan, culture-based societal, family and individual networking encourages healthy interactions. Risk-prone individuals are supported through measures such as rational and diverse discussion of news, data and information; shared meals and family gatherings to the extent that are consistent with minimum requirement of social distancing [8,9].

Our call to action seeks to mitigate, if not totally avoid, a post-COVID-19 mental health pandemic. We solicit intervention from key stakeholders such as health professionals, politicians, government, community and family members, to create awareness about potential severe mental health issues in the prevailing situation. The key stakeholders are urged to disseminate messages and optimism while creating awareness about the predicted psychological and mental health problems not only in COVID-19 patients and SARS-CoV-2 infected, but also among health workers, communities and societies. More specifically, we suggest three relatively low cost, high impact measures to be taken as soon as possible:

-  That stakeholders should use real-time virtual meeting technology to form (or mobilize already available) mental health councils. The councils or other such forums may be charged with taking active steps for mitigation of mental health fallout of the pandemic at national, regional, urban and rural levels. In this regard, information may be disseminated to the vulnerable through all possible means.
-  That such councils immediately prepare and disseminate material regarding mental health vis-à-vis the COVID-19 pandemic, expressing optimism and detailing mitigation steps consistent with national, cultural and language resources and needs. A good example has already been set by Brigham and Women’s Hospital as already stated [10].
-  That all existing social networks be activated to augment the efforts of the councils. A mechanism must be put in place to identify high-risk individuals or groups and circumstances or signs/symptoms that indicate possible risk of danger to mental health.

Finally, we urge worldwide organizations with the means and resources to approach high-risk countries and communities, to offer help to insure mental health for all.

The immediate and indispensable responses in the prevailing pandemic are to quickly and accurately test for and isolate those positive for SARS-CoV-2, provide healthcare to those with COVID-19, and to impose conditions to severely limit transmission. Unfortunately, all these responses dramatically increase conditions conducive for psychological distress. The negative impact of the isolation, distancing, lockdowns, poor healthcare and poor vaccination record will be felt more deeply and for a longer period of time in developing countries.

## Conclusion & future perspective

It has been over 2 years since the first case of COVID-19 was reported in China. The long-term mental health repercussions are still unknown and could increase demand for psychiatric therapies for several years. Mental healthcare has never been on the priority list for public health in developing counties. It is due to the COVID-19 pandemic that the importance of mental health field has been raised among people around the world. Therefore, we must consider implications for the future, in terms of mental health.

The following recommendations are put forth for enhancing the level of awareness among the general public:

- Awareness about psychological challenges during pandemics needs to be created at all levels including media platforms.
- Mental health issues should be openly debated through seminars, conferences and workshops etc. This will encourage interaction between patients, clinicians and family members etc. Similarly, such events must also be organized in schools, colleges and universities.
- The government should take responsibility and should provide the necessary facilities for dealing with mental care issues and concerns.
- The government should increase mental health funding for pandemic-related cases.
- Low-income families and those who have lost their employment, require moral and financial support from governments and authorities.
